# The impact of online health community engagement on lifestyle changes: A serially mediated model

**DOI:** 10.3389/fpubh.2022.987331

**Published:** 2022-10-03

**Authors:** Ping Zhou, Yujie Zhao, Suping Xiao, Kangsheng Zhao

**Affiliations:** ^1^Innovation and Entrepreneurship Education Research Center, Guangdong University of Foreign Studies, Guangzhou, China; ^2^School of Management, Shanghai University, Shanghai, China; ^3^School of Management, Sun Yat-sen University, Guangzhou, China; ^4^Department of Economic Management, Guangdong Construction Polytechnic College, Guangzhou, China

**Keywords:** online health community engagement, informational support, emotional support, health self-efficacy, lifestyle changes, social network theory

## Abstract

**Background:**

Due to reduced physical labor and increased food availability, making healthy lifestyle changes is becoming increasingly challenging. Prior studies have suggested that strong ties (such as friends or family members) help promote positive lifestyle behavior changes while weak ties like online friends hardly make a difference in activating healthy lifestyle changes. More recent studies have found evidence of positive lifestyle changes brought about by health APPs. Yet, the process through which online health community (OHC) engagement is related to healthy lifestyle changes has not been fully explored.

**Methods:**

Drawing on social network theory and the self-efficacy literature, we argued that the information and emotional support which users obtained from OHCs is positively associated with health self-efficacy, which in turn is positively associated with lifestyle changes. Then we constructed a serially mediated model between OHC engagement and healthy lifestyle changes and collected 320 valid questionnaires through an online survey. We tested the model by applying structural equation modeling *via* Mplus 8.3, which uses bootstrapping (5,000 samples) to test the significance of the mediated paths.

**Results:**

This study demonstrated that the informational and emotional support that users receive from OHC engagement positively affects healthy lifestyle changes *via* the mediating role of health self-efficacy. We also found that healthy lifestyle changes are an outcome of enhanced health self-efficacy through the effect of informational and emotional support from OHC engagement.

**Conclusions:**

Our findings help explain how OHC users make healthy lifestyle changes by utilizing the informational and emotional support to develop health self-efficacy. The results also highlight the value of informational and emotional support as important resources which users acquire from OHC engagement. Thus, we suggest that OHC users utilize the informational and emotional support to enhance health self-efficacy and facilitate healthy lifestyle changes. Future research could explore the dynamic process through which OHC engagement influences lifestyle changes by designing longitudinal research and addressing the limitations of the present study.

## Introduction

It has long been known that lifestyle changes are crucial in the prevention and management of chronic illnesses like cardiovascular (CVD) and coronary heart disease (1). Lifestyle improvement not only contributes to the effectiveness of chronic disease treatment but also prevents chronic diseases and delays their progression. However, due to reduced physical labor and increased food availability, making healthy lifestyle changes has become even more challenging as the instinct to prefer high sugar and fat is difficult to change (2).

Although it is not easy to make healthy lifestyle changes for many, researchers have found that socially supportive conditions help promote positive lifestyle behavior changes (3, 4). Based on social network theory, studies have suggested that maintaining a good lifestyle (such as moderately intense physical activity, a balanced diet) often requires supervision and encouragement from strong ties, which generally refer to friends or family members (5, 6). Online friends, on the other hand, are traditionally seen as weak ties (7). Yet, as the epidemic continues and information technology evolves, people are spending increasing time online, and friends in online communities may become even more intimate and trustworthy than friends and relatives in real life. This leads to a question: do information and encouragement from OHC engagement help promote lifestyle changes? OHC studies have found evidence that OHCs provide social support (i.e., informational support, emotional support, and companionship) to their users (8, 9) and social support from OHCs promote beneficial health outcomes such as weight loss (10), users' health knowledge (11), reduced uncertainty regarding the diagnosis and treatment (12), health attitude toward chronic diseases (11), and rural–urban health disparities (13). These studies suggest that social support derived from OHCs helps promote users' health literacy, attitudes, and behaviors. Yet, to our knowledge, there are few studies directly examining the relationship between OHC engagement and healthy lifestyle changes. It remains unknown whether OHC users acquire the psychological resources to make lifestyle changes. As noted by Jarbøl et al. (1), it takes not only health knowledge and attitude but also strong motivation and willpower to maintain lifestyle changes. Furthermore, although studies have suggested that OHC users' health behavioral changes are influenced by their peers in the community (14, 15), few have explored the process through which OHC engagement affects health behaviors. Understanding the mechanisms underlying the relationship between OHC engagement and health behaviors can help guide people at high risk of various diseases to make better use of OHCs and achieve sustained lifestyle improvement.

To address the gap, we developed a serially mediated model of the relationship between OHC engagement and healthy lifestyle changes drawing on social network theory, which suggests that people embedded in social networks are generally influenced by their peers' thoughts and behaviors. Our empirical test, which used 320 valid questionnaires from an online survey, found that the informational and emotional support that users receive from OHC engagement affects healthy lifestyle changes *via* the mediating role of health self-efficacy. The present study advances OHC research by explaining how OHC engagement leads to positive lifestyle changes and enriches the research on the antecedents of health self-efficacy. Moreover, this study also contributes to social network theory by exploring its validity in virtual social networks. Our findings suggest that OHC users actively utilize the informational and emotional support to enhance health self-efficacy and facilitate healthy lifestyle changes.

## Theory background and hypotheses

### Social network theory

Social network theory, which can be traced back to 1930s as a new paradigm of sociological research, becomes popular in explaining various behaviors (including individual, organization) in the network context (16, 17). A key tenet of social network theory is that people in social situations think and act in similar ways because of their relationship to each other (18). According to social network theory (19), the strength of social network relationships depends on the amount of time and energy, emotional intensity, and reciprocity that individuals invest in their social networks. Strong ties may provide individuals with emotional support and substantial help while the strength of weak ties mainly lies in heterogeneous information involved (20). Granovetter suggested that weak relationships between individuals may be a more important factor than strong relationships in influencing the attitudes and behaviors of members of a society (20). Social ties, either strong ties or weak ties, have been found to be beneficial to the physical and mental wellbeings of individuals according to many psychosocial studies (21, 22).

### Health self-efficacy

Self-efficacy was first introduced by Bandura and is defined as an individual's beliefs about his or her ability to exert control over the events that affect him or her (23). Individuals with a high level of self-efficacy are more likely to complete a given task because they are able to perform positively over a longer period, work harder in the face of challenges and difficulties, and have more specific and clear plans and strategies for accomplishing their intended goals (24). Health self-efficacy is an application of self-efficacy in the health domain and refers to people's beliefs about their ability to control what affects their health (25, 26). Individuals with a high level of self-efficacy are more likely to participate in healthy activities, which is consistent with the domain-specific nature of self-efficacy (27). Health self-efficacy has been found to be an important factor in predicting health behaviors and health outcomes.

### OHC engagement and lifestyle changes

OHCs are online platforms for users to share health experiences, post-health queries, seek, and/or offer support (28). People concerned about health issues can search for health knowledge, consult with doctors, and interact with other users to obtain or share information and experiences through OHCs. The rapid development of OHCs is important for individuals' health management and have aroused academic attention (29, 30). Many scholars have explored the sharing behavior (31), information seeking or adoption behavior (32), and continuous usage of OHC users (33) from different theoretical perspectives. For instance, Zhou et al. analyzed the antecedents of knowledge sharing intentions and behaviors of OHC users from the perspectives of community quality and social support (34). Mirzaei and Esmaeilzadeh evaluated the impacts of perceived channel richness and social exchange on patient engagement in OHCs applying the theoretical lens of social exchange theory and channel expansion theory (32). Although there have been extensive studies on the antecedents of OHC knowledge sharing and social support [e.g., (35, 36)], less work concerns what this social support from OHCs leads to. Indeed, prior studies have identified apparent and important benefits of OHCs such as informational and emotional support, yet more long-term benefits need to be further explored (37). After all, the long-term benefits for users are crucial for the value and sustainability of OHCs.

Although members of OHCs may maintain weak ties, they are more likely to exchange in-depth personal experiences and emotional support compared with traditional weak ties that formed offline due to the anonymity of virtual health communities (23). On the one hand, users who invest time and effort in browsing and participating in discussions in OHCs may gradually develop trust in other members. On the other hand, there is less concern about being judged in virtual communities as in reality. User interactions in OHCs may not only yield the benefit of heterogeneous information from weak ties but are also more likely to promote emotional support and companionship previously only derived from strong ties.

Therefore, individuals' health attitudes or behaviors may be changed because of the behaviors of other members in OHCs, which is known as peer effects (38). Peer effects among social networks have long been established and used to explain mutual influence in fields such as education, management, sociology, and finance. For example, research in education has found that the qualities and behaviors of peers are the most important factors predicting student achievement (39). Similarly, health researchers have also found that obesity is transmitted among friends and siblings, demonstrating the role of the network effect (40, 41). Moreover, Kim found that upward comparison has a significant positive effect on the self-efficacy of fitness App users (42).

Since people are inclined to present their positive images and achievements on social media, OHC users are more likely to be exposed to healthy behavior sharing, which in turn may activate their psychological energy and drive them to make positive healthy lifestyle changes. Consistent with our arguments, Anderson-Bill et al. found that communication in OHCs helps individuals gain health knowledge and emotional support, which promotes positive lifestyle changes (3). Thus, we propose:

*Hypothesis 1: OHC engagement is positively associated with healthy lifestyle changes*.

### Health self-efficacy and lifestyle changes

Many studies have found that individuals' health behaviors are influenced by the peers in their social network, yet how OHC engagement affects health behaviors should be further explored. Drawing on recent work in self-efficacy, we argue health self-efficacy plays a mediating role in the relationship between OHC engagement and healthy lifestyle changes.

Health self-efficacy—an individual's belief in his or her capabilities to manage self-health conditions—has been found to play an important role in predicting health behaviors and health outcomes. For example, Pálsdóttir found that individuals with high levels of health self-efficacy were more likely to participate in physical activity (43). Using a sample of women with heart disease, Clark and Dodge found that health self-efficacy significantly influenced people's disease management behaviors, including medication use, exercise habits, and ability to cope with stress (44). Prior studies have suggested that the higher the level of health self-efficacy, the more effective the use of health information and external support, and thus the more likely it is to make positive lifestyle changes. Thus, we propose:

*Hypothesis 2: Health self-efficacy is positively associated with healthy lifestyle changes*.

### OHC engagement and informational/emotional support

Recent studies in social psychology have showed that gaining social support is the main motivation for users to participate in online communities (6, 45). Social support refers to an individual's feeling that he or she is cared for, valued, and that his or her wellbeing is the responsibility of others in the social network (46). According to OHC literature, social support in OHCs consists of three main categories: informational support, emotional support, and companionship (47) and the social support people obtain from OHCs is very similar to the support they receive from offline social networks (45, 48). Due to the development of information technology, the links in online communities break the boundaries of time, geographic distance, and even social class (49) and allow people with similar health conditions around the world to connect and communicate. Thus, OHC users can share and seek informational and emotional support conveniently and candidly as they can use virtual identities to avoid any embarrassment or judgment. Through convenient and candid interaction (Q&A, reflection, advice-seeking, feedback), OHCs enable individuals to acquire more targeted health knowledge and emotional support. Thus, we propose:

*Hypothesis 3: OHC engagement is positively associated with informational support*.

*Hypothesis 4: OHC engagement is positively associated with emotional support*.

### Informational support, health self-efficacy, and lifestyle changes

Compared to offline visits and web searches, OHCs have unique advantages in terms of information access. First, compared with offline medical consultations, health information acquisition from OHCs is quick and relatively inexpensive (50). Second, compared with web search, OHCs break through deficiencies such as information overload, conflicting information, and lack of critical information (30, 51). Studies have found that people may become more anxious after searching online for information about diseases related to their symptoms (52). However, the experience and information offered by physician users and other OHC members with similar medical conditions has the characteristics of accuracy, relevance, and timeliness (53, 54). For instance, Kim and Mrotek found that OHCs provide up-to-date and accurate information that is not available on other websites (55). Because people tend to protect their health privacy, private health experience sharing in real life may only occur between people with solid trust rather than strangers who have just met. In contrast, the anonymity of the virtual environment facilitates the sharing of tacit and private health experience, and thus more valuable personal coping experiences are shared. Studies have demonstrated that users tend to communicate with OHC peers who have similar backgrounds (56). Therefore, OHC users are able to discuss real, concrete daily events and mental states, which helps inspire individual self-reflection and develop health strategies that are appropriate for themselves, thereby enhancing health self-efficacy (57). A study including 3,014 persons in the United States found that more than half of the population with chronic illnesses participated in OHC interactions, with 18% of all internet users reporting going online to find others who have similar medical conditions (7). The access to relevant health knowledge and experience leads to OHC users' awareness about their own health risks and possible advantages of preventive measures, which in turn may promote health self-efficacy and drive them to initiate changes.

In addition, the algorithms of OHCs can recommend more accurate content for individuals, thus helping them to obtain customized health information more easily and to receive real-time guidance from professional doctors if needed (32). Taken together, OHC engagement helps individuals access informational support and construct personalized health programs more efficiently, which positively affects health self-efficacy and healthy lifestyle changes. Thus, we propose:

*Hypothesis 5: Informational support from OHC engagement is positively associated with health self-efficacy, which in turn is positively associated with lifestyle changes*.

### Emotional support, healthy self-efficacy, and lifestyle changes

Online communities not only facilitate fast and convenient information exchange but also help individuals with previously niche needs overcome spatial and time constraints to find each other and develop supportive relationships. Through an analysis of users' fitness programs posted in OHCs, Centola, and van de Rijt found that active participants in OHCs typically selected friends by gender, age, and BMI similarity (58). Online communities reduce social costs substantially, and OHC users are able to avoid low-quality superficial socialization without embarrassment and develop high-quality relationships based on common goals, allowing users to communicate with people with similar health concerns (59). Therefore, people are more likely to gain emotional support (i.e., recognition, companionship, and attention) through OHC engagement compared with traditional ways of socializing (56, 57). Through interacting OHC members with similar experiences or the same medical condition, OHC users may gain emotional support that help them reduce the stress and/or refill psychological energy. For example, when users with weight control goals feel hungry, they may join OHCs to browse the diet diaries of users they follow to motivate themselves; they may even actively seek supervision or psychological support from other community friends with similar goals to counteract instinctive cravings for food and thus help themselves achieve health goals (60). Thus, we propose:

*Hypothesis 6: Emotional support from OHC engagement is positively associated with health self-efficacy, which in turn is positively associated with lifestyle changes*.

**Figure 1:**
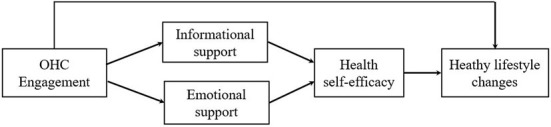
Research model.

In summary, Figure 1 shows the theoretical model summarizing the hypotheses.

## Methodology

### Sample

To test our hypotheses, we collected data through an online survey in June 2022. The online survey was conducted on Sojump (http://www.sojump.com/). We used a snowball sampling method, which presents a link directing users to the questionnaire through referrals on WeChat. Respondents were offered a small amount of money (RMB 1) to encourage participation. We received 427 questionnaires initially. After removing invalid questionnaires (i.e., where all the answers were the same or obviously contradictory), we obtained a total of 320 valid questionnaires, with a valid return rate of 74.94%. The demographic information of the sample is shown in Table 1.

**Table 1:** Sample characteristics.

**Characteristics**	**Levels**	**Frequency**	**Proportion (%)**
Gender	Male	86	26.88
	Female	234	73.12
Age	<25	313	97.82
	26–35	5	1.56
	36–45	1	0.31
	>46	1	0.31
Education	High school	4	1.25
	College	263	81.93
	Undergraduate	49	15.26
	Postgraduate	4	1.25
Marital status	Married	11	3.43
	Unmarried	309	96.57

### Measurements

We adapted measures from prior studies to test the conceptualized model. Online health community engagement was assessed with the one-item measure following Mirzaei and Esmaeilzadeh (32). The other measures, including lifestyle changes, informational support, emotional support, and health self-efficacy, use five-point Likert scales ranging from 1 “strongly disagree” to 5 “strongly agree”. To prepare the questionnaire, the authors first independently translated the English version of the questionnaire into Chinese to verify that there were no differences between the Chinese and English versions of the constructs. Next, we invited a panel of peer researchers to examine the content validity and any semantic ambiguity. Last, the Chinese version of the questionnaire went through one round of pilot testing with 5 respondents before administration of the official survey. Participants were asked to rate the items based on their own experiences or attitudes. Appendix A lists the items for the primary measures in this study.

#### Online health community engagement

Following Mirzaei and Esmaeilzadeh (32), OHC engagement was measured by the question, “How often do you use online health communities? (OHCs include Bo He Health, KEEP, Ping'an Health, Ding Xiang Doctor, Good Doctor or other health platforms). Never/ Once every few months/1–2 times a month/1–2 times a week/Almost daily.” (1 = Never, 5 = Almost daily).

#### Lifestyle changes

Lifestyle changes (LC) were measured with a three-item scale developed by Chen et al. (61). A sample item is “I am currently increasing physical activity”. Cronbach's alpha for this measure was 0.870.

#### Informational support

Informational support (IS) was assessed using a four-item scale adapted from Shirazi et al. (57). A sample item is “The information provided by the online health community meets my needs.” Cronbach's alpha for this measure was 0.937.

#### Emotional support

Emotional support (ES) was measured with a scale adapted from Johnson and Lowe (63), which contains four items such as “Other users in the OHC provide encouragement to me.” Cronbach's alpha for this measure was 0.935.

#### Health self-efficacy

Health self-efficacy (HSE) was measured with a scale developed by Oh et al. (62), which contains four items such as “I have been able to meet the goals I set for myself to improve my health.” Cronbach's alpha for this measure was 0.936.

#### Control variables

To rule out any influence from the demographic characteristics, we controlled for age, education level, gender, and marital status for the following reasons. First, existing research suggests that age is related to the difficulty of lifestyle changes (64). We categorized respondents into four groups according to their age (i.e., <25 is set to 1, 26–35 is set to 2, 36–45 is set to 3, >46 is set to 4). Second, previous research on lifestyle changes found that education level is an important antecedent for lifestyle changes (61). Thus, we controlled for respondents' education level (education was assigned the value of 1, 2, 3, and 4 for high school and below, junior college, undergraduate, graduate and above, respectively). Third, Teachman found that both gender and marital status affect individuals' lifestyle habits (65). We thus controlled for gender and marital status. Gender is assigned a value of 1 if the respondent is male, and 2 if the respondent is female. Marital status takes the value of 1 if the respondent is married or cohabited, and 2 otherwise.

## Results

### Reliability and validity

The reliability and validity of lifestyle changes, health self-efficacy, informational support, and emotional support were tested using SPSS 24.0, and the results are shown in Table 2. The KMO and AVE values are higher than 0.7, and the CR values are higher than 0.9, indicating that all the variables selected in this study have good reliability and validity.

**Table 2:** Scale properties.

**Variables**	**Items**	**Factor loading**	**Cronbach's α**	**KMO**	**CR**	**AVE**
Lifestyle changes (LC)	LC1	0.858	0.870	0.719	0.920	0.794
	LC2	0.918				
	LC3	0.896				
Health self-efficacy (HSE)	HSE1	0.819	0.936	0.864	0.904	0.703
	HSE2	0.877				
	HSE3	0.836				
	HSE4	0.821				
Informational support (IS)	IS1	0.800	0.937	0.856	0.907	0.710
	IS2	0.843				
	IS3	0.842				
	IS4	0.884				
Emotional support (ES)	ES1	0.808	0.935	0.849	0.905	0.704
	ES2	0.885				
	ES3	0.800				
	ES4	0.861				

### Common method bias and confirmatory factor analysis

First, in order to test whether there is common method bias, we used Harman's one-way test to examine common method bias on the sample data, and conducted an unrotated principal component analysis on all question items. We found that the variance explained by the first factor was 38.408% (<40%), indicating there was no serious common method bias problem that a single factor explained most of the variance. Second, to verify the structural validity of the model, we conducted a confirmatory factor analysis of all the variables using AMOS 25.0. As shown in Table 3, all the indicators meet the fit requirements, and the five-factor model achieves optimal fitness among the alternative models.

**Table 3:** Results of confirmatory factor analysis.

**Model**	**X^2^**	**Df**	**X^2^/df**	**NFI**	**CFI**	**GFI**	**RMSEA**
Model 1: one-factor model All five variables combined	1,371.166	104	13.184	0.711	0.726	0.586	0.195
Model 2: two-factor model LC + (OHC, IS, ES, HSE combined)	1,184.594	103	11.501	0.750	0.766	0.621	0.181
Model 3: three-factor model LC + HSE + (OHC, IS, ES combined)	468.985	101	4.643	0.901	0.920	0.808	0.107
Model 4: four-factor model LC + HSE + IS + (OHC and ES combined)	200.478	98	2.046	0.958	0.978	0.926	0.057
Model 5: four-factor model LC + HSE + ES + (OHC and IS combined)	192.719	98	1.967	0.959	0.980	0.929	0.055
Model 6: five-factor model LC + HSE + ES + IS + OHC	147.289	89	1.958	0.988	0.985	0.973	0.044

### Descriptive statistical analysis

Table 4 presents the means, standard deviations, and correlation coefficients between variables for all variables. The correlation analysis showed that (1) online health community participation and lifestyle improvement were significantly positively correlated (*r* = 0.256, *p* < 0.01); (2) health self-efficacy was significantly positively correlated with lifestyle improvement (*r* = 0.608, *p* < 0.01); (3) informational support was significantly positively correlated with health self-efficacy (*r* = 0.567, *p* < 0.01), and emotional support was significantly positively correlated with health self-efficacy (*r* = 0.584, *p* < 0.01). The correlation analysis provided preliminary supporting evidence for our hypotheses.

**Table 4:** Means, standard deviations, and correlations.

**Variable**	**1**	**2**	**3**	**4**	**5**	**6**	**7**	**8**	**9**
1. OHC	1								
2. IS	0.329^***^	1							
3. ES	0.277^***^	0.791^***^	1						
4. HSE	0.202^***^	0.567^***^	0.584^***^	1					
5. LC	0.265^***^	0.642^***^	0.659^***^	0.608^***^	1				
6. Gender	0.088	0.046	0.104	0.110^*^	0.037	1			
7. Age	0.204^***^	−0.010	0.011	0.064^**^	0.026	−0.026	1		
8. Edu	0.115^**^	−0.123^***^	−0.123^**^	−0.014	−0.003	−0.003	0.179^***^	1	
9. Mar	−0.021	0.068	0.050	−0.010	−0.026	−0.026	−0.224^**^	−0.234^**^	1
Mean	2.153	3.320	3.201	3.409	3.251	1.735	1.033	2.170	1.971
SD	1.067	0.815	0.824	0.830	0.853	0.446	0.278	0.451	0.182

***, **, * Denote significance levels at 1, 5, and 10%, respectively.

### Hypothesis testing

**Figure 2:**
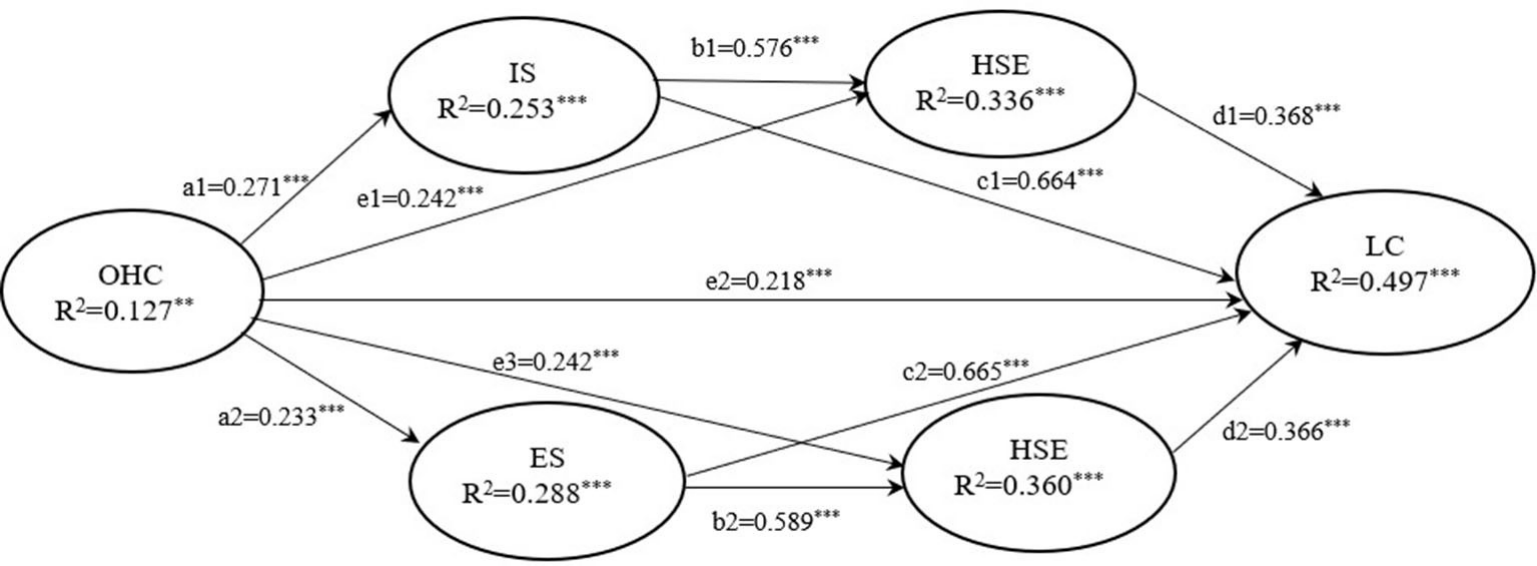
The serially-mediated relationship between OHC engagement and lifestyle changes. ***, **, *Denote significance levels at 1, 5, and 10%, respectively.

We tested the mediating effects with a Bootstrap method based on the structural equation modeling *via* Mplus 8.3. The results of the Bootstrap test and the results of the theoretical model are shown in Table 5 and Figure 2.

**Table 5:** Tests of mediation effects using bootstrapping.

		**95% confidence interval**	
**Paths**	**Indirect effects**	**LL**	**UL**
OHC → IS → HSE	0.156	0.106	0.216
OHC → IS → LC	0.180	0.124	0.244
OHC → HSE → LC	0.089	0.038	0.149
IS → HSE → LC	0.213	0.141	0.301
OHC → IS → HSE → LC	0.057	0.108	0.248
OHC → ES → HSE	0.137	0.083	0.200
OHC → ES → LC	0.155	0.096	0.220
ES → HSE → LC	0.217	0.132	0.295
OHC → ES → HSE → LC	0.051	0.092	0.227

Hypothesis 1 predicted that OHC engagement is positively associated with lifestyle changes. The path coefficient of OHC participation → lifestyle change (e2) is 0.218, *p* < 0.01, indicating that OHC participation can significantly and positively predict lifestyle change. Thus, Hypothesis 1 is supported.

Hypothesis 2 predicted that health self-efficacy is positively associated with lifestyle changes. The path coefficients (d1 and d2) of health self-efficacy → lifestyle change are 0.368 (*p* < 0.01) and 0.366 (*p* < 0.01) respectively, indicating that health self-efficacy positively predicted lifestyle change. Thus, Hypothesis 2 is supported.

Hypothesis 3 predicted that OHC engagement is positively associated with informational support. The path coefficient (a1) of OHC participation → informational support is 0.271 (*p* < 0.01), indicating that OHC participation positively predicted informational support. Therefore, Hypothesis 3 is supported.

Hypothesis 4 predicted that OHC engagement is positively associated with emotional support. The path coefficient (a2) of OHC involvement → emotional support is 0.233 (*p* < 0.01), indicating that OHC involvement positively predicted emotional support. Thus, Hypothesis 4 is supported.

Hypothesis 5 predicted that informational support from OHC is positively associated with health self-efficacy, which in turn is positively associated with lifestyle changes. The path coefficient (b1) of informational support → health self-efficacy is 0.576 (*p* < 0.01), indicating that informational support had a significant positive predictive effect on health self-efficacy. The path coefficient (d1) of health self-efficacy → lifestyle change is 0.368 (*p* < 0.01), indicating that health self-efficacy positively predicted lifestyle change. Moreover, the partial mediating effect of health self-efficacy between informational support and lifestyle change was significant (β = 0.213, *p* < 0.01), and the 95% confidence interval for Bootstrap = 5,000 is (0.141, 0.301) excluding 0. Thus, Hypothesis 5 is supported.

Hypothesis 6 predicted that emotional support from OHC is positively associated with health self-efficacy, which in turn is positively associated with lifestyle changes. The path coefficient (b2) for emotional support → health self-efficacy is 0.589 (*p* < 0.01), indicating a significant positive predictive effect of emotional support on health self-efficacy. The path coefficient (d2) of health self-efficacy → lifestyle change is 0.368 (*p* < 0.01), indicating that health self-efficacy is positively associated with lifestyle change. Moreover, the partial mediating effect of health self-efficacy between emotional support and lifestyle change is significant (β = 0.217, *p* < 0.01), and the 95% confidence interval for Bootstrap = 5,000 is (0.132, 0.295) excluding 0. Thus, Hypothesis 6 is supported.

To evaluate the statistical significance of the mediated paths, the structural equation modeling *via* Mplus 8.3 (which uses bootstrapping, 5,000 samples) was used to test the serial mediation model in Hypotheses 5 and 6. The empirical tests, using 320 valid questionnaires collected through an online survey, showed that the informational and emotional support that users receive from OHC engagement affects healthy lifestyle changes *via* the mediating role of health self-efficacy. Our serially mediated model was approved.

In addition, consistent with Teachman (65), the results show that gender is positively associated with health self-efficacy and lifestyle changes. Females have higher levels of health self-efficiency and more lifestyle changes. Age was positively associated with self-efficacy but negatively associated with lifestyle changes, suggesting that older people have higher levels of health self-efficacy but fewer lifestyle changes. Education level is positively associated with lifestyle changes, indicating that the higher the education level, the more significant the lifestyle changes, consistent with the findings of Chen et al. (61).

## Discussion

Drawing on social network theory and the self-efficacy literature, we argued that the informational and emotional support which users obtained from OHC engagement is positively associated with health self-efficacy, which in turn is positively associated with lifestyle changes. Our empirical study, which analyzed 320 valid questionnaires collected on an online survey, supported the arguments. This study demonstrated that the informational and emotional support that users receive from OHC engagement affects healthy lifestyle changes *via* the mediating role of health self-efficacy. Since scholars have focused largely on the antecedents of OHC knowledge-sharing and other forms of participation, our study advances the extant literature on the outcomes of OHC engagement.

### Theoretical contributions

This study makes three contributions to the extant research. First, our study enriches the OHC literature. Although prior OHC research has demonstrated that OHC users can receive informational support and emotional support from community interactions, more long-term benefits to users need to be further investigated. Our study examines the impact of OHC engagement on healthy lifestyle changes, thus contributing to the OHC. Moreover, through constructing a serially mediated model, our study helps explain how OHC engagement leads to positive lifestyle changes, echoing the call from Prochnow and Patterson (4).

Second, this study enriches the health self-efficacy literature by examining the effect of OHC engagement on health self-efficacy. Compared to the research on the consequences of health self-efficacy, the body of literature on the antecedents of health self-efficacy is relatively thin. By investigating the relationship between OHC engagement and health self-efficacy and the mediating roles of informational and emotional support, our results showed that informational support and emotional support are positively associated with health self-efficacy, thus contributing to the health self-efficacy literature.

Third, our study extends the application of social network theory to virtual social networks. While previous social network studies have tended to consider online friends as weak ties, this study found a positive effect of OHC engagement on users' health self-efficacy and lifestyle changes, suggesting the strength of online community peers (e.g., in-depth information-sharing and emotional support), which challenges the viewpoint that social ties formed in virtual communities generally function as weak ties to mainly provide diversified experiential information (7). Our study develops a better understanding of the role of virtual networks, thus complementing the research of social networks. Moreover, our study demonstrated that the underlying mechanism through which OHC engagement affects healthy lifestyle changes was informational support and emotional support, thus enriching social network theory in terms of its effectiveness in virtual networks.

### Practical implications

Promoting individual health management with digital technologies is a hot topic in public health literature, and our study suggests some practical implications by examining the role of OHC engagement in making healthy lifestyle changes.

First, our findings showed that the informational and emotional support is important mediating mechanisms for OHC users to make lifestyle changes. Therefore, through purposeful engagement in OHCs (i.e., gaining high-quality, highly relevant information and/or emotional support through interaction with other users), OHC users may enhance health self-efficacy and facilitate healthy lifestyle changes. Especially when individuals feel low on energy and stressed, they may browse informative and inspirational posts by other positive OHC users and proactively seek help from OHC peers to obtain health knowledge and phycological energy and maintain a healthy lifestyle.

Second, our results show that the informational and emotional support which users acquired from OHC engagement is important antecedents of health self-efficacy and healthy lifestyle changes. Thus, the platform managers of OHCs could make incentive rules to encourage users to exchange more valuable informational and emotional support to each other, so as to inspire them to achieve their health management goals. For example, in-depth personal experience sharing (e.g., disease-related diagnosis and recovery experience) is rewarded with community points and thank-you messages that recognize the value of members' positive engagement. Additionally, platform administrators of OHCs may develop a warm, trusting, inclusive community culture and hold events to promote community cohesiveness so that users can discuss their health issues openly and honestly and seek emotional support from other users without concerns of being judged. To be specific, OHC managers may employ human or automatic moderators to promote constructive sharing and eliminate unfriendly content that inhibits a cooperative community atmosphere in the OHC as suggested by James et al. (28). Moreover, OHC managers may optimize algorithms that allow users with similar health conditions to find each other and benefit from highly relevant health experience and emotional support.

Third, physicians could guide their patients to use OHCs to improve patient compliance with lifestyle behavior changes. As OHCs can provide users with informational and emotional support in an efficient way, patients using OHCs may have a higher level of health self-efficacy and less difficulty in maintaining lifestyle changes that physicians advise. If physicians could collaborate with OHCs in encouraging healthy lifestyle changes among patients with higher risks of chronic diseases, many medical resources could be saved.

### Limitations and future directions

With its theoretical and practical implications, the study has some limitations which should be considered by future studies. First, this study used cross-sectional data, which makes it difficult to rigorously examine the dynamic process of OHCs' impact on lifestyle changes. Future studies may consider adopting a longitudinal method to explore the mechanism through which OHC engagement influence lifestyle changes. Second, this study used self-reported data to measure healthy lifestyle changes, and respondents may be influenced by social desirability and conceal the truth. Therefore, future research may fruitfully attempt to measure lifestyle changes with more precise data through multiple sources. For example, collecting data from wearable devices can directly measure the volunteers' lifestyle improvement. Third, the sample coverage was inadequate, as the data mainly consists of young people, which also limits the generalization of our findings to other age groups. Future studies may attempt to confirm the results with data from different age groups.

## Data availability statement

The raw data supporting the conclusions of this article will be made available by the authors, without undue reservation.

## Author contributions

PZ: conceptualizing the research idea and writing–original draft. YZ: revising the paper. SX: processing data and visualizing. KZ: data-collecting and providing revised advice. All authors contributed to the article and approved the submitted version.

## Funding

This study was funded by the National Natural Science Foundation of China (Nos. 71802101 and 72002052), Shanghai Pujiang Program (No. 21PJC058) and Jiangxi Province Education Department (Nos. GJJ181483 and GJJ200507).

## Conflict of interest

The authors declare that the research was conducted in the absence of any commercial or financial relationships that could be construed as a potential conflict of interest.

## Publisher's note

All claims expressed in this article are solely those of the authors and do not necessarily represent those of their affiliated organizations, or those of the publisher, the editors and the reviewers. Any product that may be evaluated in this article, or claim that may be made by its manufacturer, is not guaranteed or endorsed by the publisher.

## APPENDIX A Measurement scales

**Table:**

**Construct**	**Items**	
Lifestyle changes (LC)	LC1: I am currently increasing physical activity	(61)
	LC2: I am Currently reducing fat/calories in my diet	
	LC3: I am Currently participating in weight control	
Health self-efficacy (HSE)	HSE1: I am confident I can have a positive effect on my health	(62)
	HSE2: I am actively working to improve my health	
	HSE3: I have set some definite goals to improve my health	
	HSE4: I have been able to meet the goals I set for myself to improve my health	
Informational support (IS)	IS1: I will ask others for help with health information through the online health community	(57)
	IS2: Other users provide good advice based on my request for help	
	IS3: The information provided by the online health community meets my needs	
	IS4: I take my health more seriously because I participate in the online health community	
Emotional support (ES)	ES1: Other users in the community understand my situation	(63)
	ES2: Other users in OHC show me empathy	
	ES3: Other users in OHC provide encouragement to me	
	ES4: I feel a sense of connectedness when I see posts from the community	
OHC Engagement (OHC)	How often do you use online health communities?	(32)
	Never/ Once every few months/ 1-2 times a month/ 1-2 times a week/ Almost daily	
